# Vancouver At Home: pragmatic randomized trials investigating Housing First for homeless and mentally ill adults

**DOI:** 10.1186/1745-6215-14-365

**Published:** 2013-11-01

**Authors:** Julian M Somers, Michelle L Patterson, Akm Moniruzzaman, Lauren Currie, Stefanie N Rezansoff, Anita Palepu, Karen Fryer

**Affiliations:** 1 Somers Research Group, Faculty of Health Sciences Simon Fraser University, 8888 University Drive, Burnaby V5A 1S6, Canada; 2 Department of Medicine, University of British Columbia, 2775 Laurel Street, Vancouver V5Z 1M9, Canada

**Keywords:** Housing First, Homelessness, Mental illness, Concurrent disorders

## Abstract

**Background:**

Individuals with mental illnesses are overrepresented among the homeless. Housing First (HF) has been shown to promote positive outcomes in this population. However, key questions remain unresolved, including: how to match support services to client needs, the benefits of housing in scattered sites versus single congregate building, and the effectiveness of HF with individuals actively using substances. The present study aimed to recruit two samples of homeless mentally ill participants who differed in the complexity of their needs. Study details, including recruitment, randomization, and follow-up, are presented.

**Methods:**

Eligibility was based on homeless status and current mental disorder. Participants were classified as either moderate needs (MN) or high needs (HN). Those with MN were randomized to HF with Intensive Case Management (HF-ICM) or usual care. Those with HN were randomized to HF with Assertive Community Treatment (HF-ACT), congregate housing with support, or usual care. Participants were interviewed every 3 months for 2 years. Separate consent was sought to access administrative data.

**Results:**

Participants met eligibility for either MN (n = 200) or HN (n = 297) and were randomized accordingly. Both samples were primarily male and white. Compared to participants designated MN, HN participants had higher rates of hospitalization for psychiatric reasons prior to randomization, were younger at the time of recruitment, younger when first homeless, more likely to meet criteria for substance dependence, and less likely to have completed high school. Across all study arms, between 92% and 100% of participants were followed over 24 months post-randomization. Minimal significant differences were found between study arms following randomization. 438 participants (88%) provided consent to access administrative data.

**Conclusion:**

The study successfully recruited participants meeting criteria for homelessness and current mental disorder. Both MN and HN groups had high rates of substance dependence, suicidality, and physical illness. Randomization resulted in no meaningful detectable differences between study arms.

**Trial registration:**

Current Controlled Trials: ISRCTN57595077 (Vancouver at Home study: Housing First plus Assertive Community Treatment versus congregate housing plus supports versus treatment as usual) and ISRCTN66721740 (Vancouver At Home study: Housing First plus Intensive Case Management versus treatment as usual).

## Background

Individuals who are homeless and mentally ill are heterogeneous in their health and social challenges. Effective models of service must be responsive to individual needs, which may vary across time and space, and are constrained by pragmatic factors, including local standards of care, housing availability, and funding. The Vancouver At Home (VAH) project has implemented two randomized controlled trials (RCTs) involving homeless mentally ill adults in Vancouver, BC, Canada. VAH is collaborating with similar projects in four other Canadian cities [1]. Each collaborating center has incorporated a common methodology, with pragmatic adaptations in each site. Site-specific adaptations were influenced by the characteristics of each local population (for example, ethno-racial services in Toronto, ON, Aboriginal focus in Winnipeg, MB), as well as the structural features of each locale (for example, rural service models in Moncton, NB, congregate housing in Vancouver, BC). The purpose of the present article is to describe the unique features of VAH, including measures, interventions, and sample characteristics in accordance with the Consolidated Standards of Reporting Trials (CONSORT) statement for the reporting of pragmatic trials.

We briefly describe the physical setting of Vancouver, the local population who are both homeless and mentally ill, and the Housing First (HF) program. These factors influenced both the design and implementation of VAH with the goal of maximizing the effectiveness and relevance of the project.

For decades, the city of Vancouver has struggled to meet the needs of a visibly homeless and inadequately sheltered population in a central downtown neighborhood. The same neighborhood has been afflicted by high crime rates, an open market for illicit drugs, infectious diseases, and premature mortality [2]. For many years, the most affordable housing in the neighborhood has consisted of single room occupancy (SRO) hotels, many of which earned a reputation for hazards ranging from bed bugs to criminal predation [3,4]. Individuals with mental illness are prominent among the homeless, particularly following 'deinstitutionalization’, whereby regional psychiatric facilities were downsized as promises to implement community-based support, such as Assertive Community Treatment (ACT), were unfulfilled. A diverse array of services emerged over time to support individuals in the neighborhood, including outreach services, meal programs, shelters, drug-related services (for example, needle exchange, supervised injection site [5]), and varied forms of supported housing.

The City of Vancouver has implemented a plan with the goal to 'end street homelessness’ by 2015. A key element of the plan involves the construction of apartment buildings to provide housing and support for the homeless. However, key questions remain unresolved regarding the appropriate mix of occupants in these buildings and the type of support that would be required to promote stable occupancy and recovery among individuals who are leaving homelessness and who have differing needs.

Prior to VAH, no study had systematically examined the health and housing status of individuals who were homeless and mentally ill in Vancouver. A considerable amount of anecdotal and descriptive information was available from sources, such as shelter operators, street outreach clinicians, police, and from research involving samples that included homeless individuals (for example, patients with HIV/AIDS, survival sex workers [4,6,7]). The available evidence suggests that the local population of homeless mentally ill individuals struggles with complex social and medical problems, including infectious diseases, frequent polysubstance use, cognitive impairment, trauma, victimization, and poor food security [4,8]. It had also been reported that homeless individuals were using emergency and hospital services due to inadequate community care, and were frequently involved with the justice system [7,9]. Based on these considerations, it was anticipated that VAH would extend the HF model to clients with more complex needs than those described in previous trials, including participants with concurrent substance use disorders.

A growing literature supports the effectiveness of the HF model for individuals who are both homeless and mentally ill. HF emphasizes the value of client choice and has been shown to promote residential stability [10,11], community integration [12], and high levels of client satisfaction [13]. Originating from the Pathways model in New York City, NY, USA, HF involves building a portfolio of rental accommodations (typically apartments) scattered throughout different neighborhoods, thereby providing clients with meaningful choices concerning the location and setting of their residence [14]. Clients are then supported in their homes by either an HF with Assertive Community Treatment (HF-ACT) team or HF with Intensive Case Management (HF-ICM), depending on their level of needs. ACT was originally created to constitute a 'hospital without walls’ enabling individuals who might otherwise have been admitted to psychiatric facilities to instead pursue recovery in community settings [15]. ACT teams are available 24/7, and include varied expertise across multiple disciplines. The effectiveness of ACT has been well established among individuals who reflect the original target population (that is, individuals with psychotic disorders or bipolar disorder), including specific outcomes, such as reductions in hospital admissions and criminal justice involvement [16,17]. However, the effectiveness of HF-ACT is less well known among sub-populations who also have cognitive impairments, complex addictions, or multiple physical and mental illnesses.

The majority of studies examining HF have followed participants for up to 24 months (for example, Gulcur [11], Tsemberis and Eisenberg [14]). Longer-term research involving diverse residential interventions for homeless individuals in New York City found that housing stability decreased after 1, 2, and 5 years (75%, 64%, and 50%, respectively) [18]. Several studies have reported that substance use disorders are predictive of lower housing stability regardless of residence type [19,20]. Preliminary evidence suggests that congregate HF (that is, a single supported building) may achieve housing stability and cost savings among homeless men who are alcohol dependent [21]. However, it is not known whether these results would be replicated among mentally ill users of illicit (or multiple) substances and who receive scattered site HF.

Compared to ACT, the effectiveness of ICM has received less empirical attention in the context of supported housing for individuals with mental illness, and its definition varies widely [22]. Unlike HF-ACT, which provides a broad range of specialized services directly to clients, HF-ICM operates as a liaison connecting clients with community services based on their expressed needs. The success of HF-ICM is therefore a function of the complexity of client needs, as well as the availability and appropriateness of relevant community resources. HF-ICM may be appropriate for clients with less severe mental illness or as a step-down from HF-ACT following successful stabilization. For example, as part of a multicenter trial, the Boston McKinney study [22] randomized homeless adults with mental illness to independent apartments or small group homes, both of which received 'comprehensive case management’. Findings indicated that housing availability, regardless of type, was the primary predictor of subsequent ability to avoid homelessness, while enhanced services reduced the risk of homelessness if housing was also available. Substance abuse was the strongest single predictor of days homeless [22,23].

The primary objectives of this article are:

1. To report study details including measures and interventions that are unique to VAH.

2. To present details of recruitment and follow-up rates for participants in VAH including primary data collection and administrative data.

3. To present baseline characteristics and examine potential non-equivalence between randomization arms in each trial.

4. To examine differences in the complexity of needs between participants in the two trials.

## Methods

### Community engagement

Development of the research protocol was preceded by several community meetings and by six focus groups with individuals who had experienced homelessness and mental illness in the Vancouver area. In total, 58 individuals were convened with the assistance of community agencies, and met privately with an experienced academic facilitator who took notes and prepared reports of proceedings. Focus group participants were asked to advise on procedures to enhance the relevance of the research, to minimize risks and maximize benefits to participants, and ways to incorporate the expertise of individuals with direct experiences of homelessness in the research project. Narrative feedback from respondents (for example, amounts of honoraria, the need to include individuals who had experienced homelessness as members of service teams and on the research team) were included in the grant application and later implemented as part of the project. Service provider representatives were consulted extensively during the design of the research and were invited to respond to a request for proposals to implement the major services that comprised the interventions tested in the study: HF-ACT, HF-ICM, scattered site housing portfolio management, and congregate housing with support.

### Recruitment

Participants were recruited through service providers and agencies serving individuals who are homeless and mentally ill in Vancouver, including shelters, drop-in centers, street outreach workers, hospitals, police, and courts. An effort was made to locate individuals throughout Vancouver, while recognizing that the majority of visible homelessness and related services were concentrated in one area.

### Eligibility and level of needs

Eligible participants were Canadian citizens at least 19 years of age who met criteria for homelessness or precarious housing and current mental disorder status. Informed consent required that individuals were made aware that randomization would involve assignment to either a pre-specified intervention that included housing or to usual care consisting of existing services and support. Participants and interviewers were therefore not blinded to the results of randomization.

### Operational definitions

Homelessness was defined as having no fixed place to sleep or live for more than 7 nights and little likelihood of obtaining accommodation in the coming month. Precarious housing was defined as currently residing in marginal accommodation, such as a SRO hotel, and having two or more episodes of homelessness (as defined above) during the past 12 months. These were minimal criteria, and participants with more long-standing homelessness were eligible for inclusion. Current mental illness was assessed using the Mini-International Neuropsychiatric Interview (MINI) [24] for the following: major depressive episode, manic or hypomanic episode, post-traumatic stress disorder, mood disorder with psychotic features, and psychotic disorder. Where possible, mental disorder status was corroborated by physician diagnosis. Participants were categorized as moderate needs (MN) or high needs (HN). Inclusion in the HN study was based on Multnomah Community Ability Scale (MCAS) [25] score of 62 or lower and current bipolar or psychotic disorder, as well as one of the following: legal involvement in the past year, substance dependence in the past month, and two or more hospitalizations for mental illness in any one of the past 5 years. All other eligible participants were included in the MN study.

### Retention strategies

A team of full-time and part-time field interviewers was recruited to follow participants at 3-month intervals. Interviewers received in-depth training and supervision in the administration of measures, and scales and items were pre-tested with a sample of participants. Interviews were considered 'on time’ if they occurred within 2 weeks of the designated anniversary date. Participants were paid C$35 for the baseline interview and approximately C$30 for each subsequent interview. Scales were administered verbally and responses entered immediately on laptop computers. Major interviews conducted at 6-month intervals required between 90 to 180 minutes to complete in most cases. A field research office was open daily throughout the study period, and participants were encouraged to drop-in regardless of their interview schedule. Interviewers obtained periodic updates regarding participants’ routines and typical whereabouts, and collateral contact information was sought in order to aid with relocation. Interviews were conducted in various locations based on randomization arm and participant preference, including participants’ homes, field research office, and public settings, such as restaurants, parks, and drop-in centers.

### Randomization

After establishing eligibility for either the MN or HN study, a computerized adaptive randomization procedure was followed to assign participants to study arms. Interviewers used laptop computers with secure live connections to upload data and receive randomization results prior to notifying participants of the outcome. Sample sizes of 100 participants in each study arm were derived based on effect size estimates of 0.5 for the major outcome variables, power of 0.80 (β = 0.20), an attrition rate of 40%, and significance levels of 0.05 (two-tailed). Additional details of sample size estimates are reported separately [1]. Based on their first 30 clients, the HF-ACT team determined that they would be able to support no more than 90 clients, and the upper limit of this arm was revised accordingly.

### Measures

VAH researchers collaborated with investigators in four other study centers and the study funder to develop a common battery of repeated measures. In addition, a number of site-specific measures were implemented at different intervals during the study (Table 1). Both shared and site-specific measures were selected based on the review of existing literature and toward addressing major gaps in knowledge. Measures were administered at single time points if their results were historical or highly stable (for example, adverse childhood events). Repeated measures (for example, quality of life, community integration) were hypothesized to be subject to variation over time based on the randomization arm, with superior outcomes in experimental conditions compared to treatment as usual (TAU). We similarly hypothesize that the models of service introduced through the study would cause superior outcomes when compared to TAU on measures of hospitalization, emergency department visits, and justice system involvement.

**Table 1:** Vancouver At Home (VAH) questionnaire details

**Acronym**	**Full name**	**Timeline**	**Key domain/topics**	**References**
ACC	Health service access items	BL, 6, 12, 18, 24	Use of community services: regular family physician or health clinic use, and perceived unmet healthcare needs.	[26,27]
ACE	Adverse Childhood Experiences	18	Psychiatric and physical health, traumatic early life events.	[28]
CI	Cognitive impairment	6, 24	Level of independent community functioning, psychiatric and physical health: Hopkins Verbal Learning Test, Trail Making Test, and Digit Symbol Test.	[29-32]
CIS	Community integration scale	BL, 6, 12, 18, 24	Level of independent community functioning, quality of life: community participation and sense of belonging.	[33-35]
CSI	Colorado Symptom Index (modified)	BL, 6, 12, 18, 24	Psychiatric and physical health: frequency of past month’s psychiatric symptoms.	[36-39]
CTS^1^	Conflict tactics scale	24	Level of independent community functioning: frequency and severity of interpersonal conflict.	[40]
CMC	Comorbid conditions list	BL	Psychiatric and physical health: presence of chronic and infectious diseases.	[41]
C-SSS	Core service satisfaction scale	24	Quality of life, use of community services: participant satisfaction with services provided by Vancouver At Home (VAH) intervention teams.	[42,43]
DSHH	Demographics, housing, vocational, and service use history	BL	Housing status, use of community services: sociodemographic details, lifetime duration of homelessness, long-term health, social and justice service use, and vocational history.	[44]
EQ-5D	EuroQol 5D	BL, 6, 12, 18, 24	Psychiatric and physical health, health-related quality of life.	[45-47]
FS	Social support items and food security	BL, 6, 12, 18, 24	Use of community services, psychiatric and physical health: type, quality, availability and source of food, and recent history of food insecurity.	[48,49]
GAIN-SPS	Global Appraisal of Individual Needs, Substance Problem Scale	BL, 6, 12, 18, 24	Substance-related problems.	[50,51]
HSJSU	Health, social, and justice service use inventory	BL, 6, 12, 18, 24	Use of community services, psychiatric and physical health: nature and frequency of health, social, and justice system services.	[42,52-57]
III	Interviewer impression items	BL, 3, 6, 9, 12, 15, 18, 21, 24	Level of independent community functioning: interviewer assessment of validity and reliability of self-report data.	
LR	Landlord relations	18	Housing status: specific to landlord relationship.	
PHQL	Perceived housing quality	6, 12, 18, 24	Housing status: subjective housing quality assessed by participants.	[58,59]
MCAS	Multnomah Community Ability Scale	BL, 6, 12, 18, 24	Level of independent community functioning: interviewer assessed level of functioning across range of domains.	[25,60]
MINI	Mini-International Neuropsychiatric Interview	BL	Psychiatric and physical health: current major Axis I disorders and suicidality.	[24,61-63]
MH	Mobility history	21	Housing status: geographic mobility.	
MoCA^1^	Montreal Cognitive Assessment	21	Level of independent community functioning: assessment of cognitive domains indicated for the screening of neurological deficits.	[64]
OHQS	Observer-rated Housing Quality Scale	24	Housing status: objective ratings of physical characteristics of participant dwellings.	
PAIN^1^	Chronic pain screener	21	Quality of life, psychiatric and physical health.	
QoLI-20	Quality of Life Index, 20-item	BL, 6, 12, 18, 24	Quality of life, psychiatric and physical health: subjective quality of life across range of domains.	[65-67]
RAS-22	Recovery Assessment Scale, 22-item	BL, 24	Quality of life, psychiatric and physical health.	[68-70]
RTLFB	Residential Time-Line Follow-Back	3, 6, 9, 12, 15, 18, 21, 24	Housing status: detailed chronology of housing status, including frequency of moves, type of accommodation, and household composition.	[39,71]
SCNR	Eligibility screening instrument	BL	Housing status, psychiatric and physical health: determines participation eligibility based on legal adult status (>19 years in British Columbia (BC)), absolute homelessness or precarious housing status, and current mental illness.	[24,61-63]
SF-12	SF-12 Health Survey	BL, 6, 12, 18, 24	Psychiatric and physical health, level of independent community functioning: assessment of the extent of impairment caused by both physical and mental illness.	[45,72,73]
VFC^1^	Foster care history	12	Quality of life, psychiatric and physical health: details early life involvement in the child welfare system.	
VMAP^1^	Maudsley Addiction Profile	BL, 6, 12, 18, 24	Psychiatric and physical health: past month’s substance use, including drug type, mode of administration, frequency of use, and drug-related harms.	[74]
VTLFB	Vocational Time-Line Follow-Back	3, 6, 9, 12, 15, 18, 21, 24	Level of independent community functioning: detailed chronological recent history of paid work and educational or skills training. Quantifies income and income sources.	[75]
WAI-PAR	Working Alliance Inventory, participant	6, 12, 18, 24	Use of community services and quality of life: participant (WAI-PAR) and service provider (WAI-PRO) assessment of working relationship with key service provider. Assessment of perception of client-provider relationship, support, confidence, and trust.	[76-80]
WAI-PRO				
	Working Alliance Inventory, provider	12		

^1^Vancouver site-specific scales. Timeline: BL, baseline; 3, 3-month visit; 6, 6-month visit; 9, 9-month visit; 12, 12-month visit; 15, 15-month visit; 18, 18-month visit; 21, 21-month visit; 24, 24-month visit.

The domains addressed by the cross-site scales are: housing and vocational status, psychiatric and physical health, level of independent community integration and functioning, quality of life, and use of community services. The following questionnaires were administered at baseline and at 6-month intervals: health service access items (ACC) [26,27]; community integration scale (CIS) [33]; Colorado Symptom Index (modified) (CSI) [36]; EuroQol 5D (EQ-5D) [45]; Global Appraisal of Individual Needs, Substance Problem Scale (GAIN-SPS) [50]; SF-12 Health Survey (SF-12) [72]; Quality of Life Index, 20-item (QoLI-20) [65]; social support items and food security (FS); health, social, and justice service use inventory (HSJSU); and MCAS [25]. Two instruments were administered at 3-month intervals, Residential Time-Line Follow-Back (RTLFB) [71] and Vocational Time-Line Follow-Back (VTLFB) [75], to produce a continuous timeline of housing status and vocational status, respectively. The Recovery Assessment Scale, 22-item (RAS-22) [68] was administered at baseline and 24 months, and the Adverse Childhood Experiences (ACE) questionnaire [28] was administered once at 18 months after baseline. A number of other measures were implemented at a single time-point: comorbid conditions list (CMC) [41], landlord relations (LR), Observer-rated Housing Quality Scale (OHQS), mobility history (MH), and core service satisfaction scale (C-SSS) [42,43]; or at other intervals: cognitive impairment (CI) (Hopkins Verbal Learning Test, Trail Making Test, Digit Symbol Test) [29-32], perceived housing quality (PHQL) [58,59], and Working Alliance Inventory (WAI) [76-80]. Semi-structured narrative interviews were scheduled at baseline and 18-month time points with approximately fifty participants (10 from each study arm in the two VAH trials).

Site-specific measures were selected based on study hypotheses and the anticipated characteristics of the Vancouver homeless population. Major areas of hypothesis testing were: addictions, cognitive impairment, and psychiatric severity would negatively influence housing stability; HF would result in superior outcomes when compared to TAU, including reduced use of crisis services and justice system encounters, superior housing stability, and quality of life; and HF would produce superior health outcomes compared to TAU.

The Maudsley Addiction Profile (MAP) [74] is a multidimensional instrument assessing alcohol and drug use and related harms, administered at 6-month intervals. The Montreal Cognitive Assessment (MoCA) [64] assesses several cognitive domains and is indicated for the screening of neurological deficits in younger populations (for example, traumatic brain injury, brain tumors, vascular cognitive impairment). The foster care history (VFC) was administered once at 12 months after baseline. The MoCA, conflict tactics scale [40], and pain scales (assessing acute and chronic pain; Schutz, unpublished) were administered at 21 months only.

Ten participants in each study arm (n = 50) were invited to participate in open-ended, qualitative interviews planned for baseline and 18 months after recruitment. Participants were selected purposively in order to represent differences across gender, ethnicity, duration of homelessness, and degree of functional impairment. Interview questions were organized around the following themes: pathways into and out of homelessness; high, low, and turning points in life; and challenges and enabling factors related to recovery.

In addition, fifty participants were asked to provide consent to undergo physical health examinations involving basic physician assessment and blood analysis (for example, hepatitis B/C, HIV/AIDS). These assessments were included to examine the possibility of undetected illness among members of the study cohorts. Finally, all participants were asked to provide consent for the researchers to send their identifying details to public agencies in order to then receive administrative data regarding their use of health, justice, and social welfare services (separate consent was sought for each category of agency). An inter-agency data sharing protocol was created by a prior project and was used as the basis for the current data extract. The fields of data specified for inclusion were: physician services; hospital services; pharmaceutical services; community mental health and substance use services; vital statistics; justice events, including convictions and sentences; and financial assistance.

### Interventions

Participants in both the MN and HN studies were randomized to either an intervention based on the principles of HF or to TAU. In both studies, TAU participants did not receive any housing or support services through the study, but were able to access existing services and support for individuals who are homeless and mentally ill in Vancouver. The resources comprising TAU include shelters, SRO hotels, and community services described earlier. No research has previously examined the responsiveness and effectiveness of these services by following a cohort of homeless individuals prospectively. Coincident with this study, the City of Vancouver and the provincial housing authority were in the process of expanding services for the homeless [81]. The quality and type of housing received by participants in TAU will be carefully documented alongside the receipt of services and support attendant to housing. In addition to TAU, HN participants were randomized to either HF-ACT or Congregate Housing with Support (CONG). MN participants received either TAU or HF-ICM.

Service providers for each intervention were selected through a competitive request for proposals. Applications were reviewed by a panel of senior individuals drawn from homelessness research, management of services, and community granting agencies. The criteria for assessment included the delineation of organizational experience, plans for implementation, and budget. Each selected service provider received specific training in the delivery of HF, and underwent fidelity assessments by external review teams at two points during the study (see below). Services were based on the model defined by Pathways to Housing [82], including expertise that anticipated the needs of local clients (for example, addiction severity), and configured to support participants in both scattered and congregate housing configurations. Participants randomized to HF were transitioned to a case manager within 2 days of study recruitment.

An inventory of apartments was developed in a variety of neighborhoods throughout the city. These apartments were drawn from private market rentals with numerous landlords. In order to promote community integration, a maximum of 20% of the units in any building could be allocated to program participants. Consistent with the principles of HF, participants were provided with a choice of housing units [82]. A housing portfolio manager was responsible for building and maintaining relationships with landlords, including relocating participants to more suitable locations when needed. Participants in the scattered site conditions (HF-ACT and HF-ICM) received support in their homes and were expected to meet with program staff on a weekly basis. The CONG condition was mounted in a single vacant hotel with the capacity to house approximately 100 occupants in independent suites but without full kitchens. The building was located in a mixed residential and commercial neighborhood, adjacent to numerous amenities. The building was equipped with a number of facilities to support residents and to promote the development of a positive community culture, including: central kitchen and meal area, medical examination room and formulary, and recreational areas (yoga, basketball, road hockey, lounge). Tenants were provided with opportunities to engage in part-time work both within the building (for example, meal preparation, laundry) and in the community (for example, providing a graffiti removal service). A reception area and front desk were staffed 24 hours a day. Tenancy in any of the experimental housing conditions was not contingent on compliance with specific therapeutic objectives (for example, addiction treatment). Program staff in each intervention condition participated in a series of training events in person in order to enhance consistency in practices. Subsidies were provided through the study to ensure that participants paid no more than 30% of the total income on rent. Fidelity assessments were conducted by an external team, with representatives from Pathways to Housing, the study funder, and individuals who had experienced homelessness. Assessments were conducted at two time points (12 and 24 months after implementation) using a HF fidelity scale [82], and involved meetings with staff as well as participants in each of the HF interventions. The assessment team provided verbal and written feedback to the staff at each intervention.

### VAH outcomes

The primary outcome domains for both trials are: housing stability, health status, quality of life, and service use. Secondary outcome domains are: cost avoidance and cost effectiveness. Primary outcomes will be compared between HF and TAU, including examination of similarities and differences between congregate and scattered site configurations of HF in the HN sample. Previous research has reported greater reductions in homelessness with group housing than with placement in independent apartments [22]. Particular attention will be paid to the role of substance use in relation to primary outcomes. Service use outcomes and economic analyses will be conducted using administrative data sources as specified.

### Data collection and analysis plan

Repeated measures (3- and 6-month scales) were collected over 24 months. Based on previous studies and the results reviewed above, approximately 2 years of follow-up was regarded as sufficient to detect changes in the major outcome domains. Fifteen months after commencing recruitment, the cross-site protocol was shortened from 24 to 21 months. Following this change, VAH revised the composition of the 21-month interview in order to provide more complete data for cross-site end point analyses; however, VAH continued to collect data through to 24 months and preserved the original protocol as specified in the trials’ registration. Differences between sites in the protocol change were primarily due to differences in financial resources between study centers. Preserving the original slightly longer follow-up in Vancouver was deemed important owing to the high levels of comorbidity, substance dependence, and chronicity of homelessness within the sample.

Descriptive statistics (such as mean, median, standard deviation, and proportions) were calculated for all quantitative instruments administered at baseline. Comparisons of variables between groups were conducted using parametric tests (*t*-tests or one-way analysis of variance (ANOVA) for continuous variables) and non-parametric tests (Mann–Whitney or Kruskal–Wallis test for continuous variables; Pearson’s chi-squared or Fisher’s exact tests for categorical variables), as appropriate. All reported *P* values were two-sided.

Longitudinal analyses of VAH data were planned for 12 and 24 months using several analytic methods, such as hierarchical linear modeling (HLM), generalized estimating equations (GEE), and time-to-event analysis (such as Cox regression and negative binomial regression), as appropriate. All longitudinal analyses are based on intention-to-treat. The major domains of longitudinal analysis examine the overall robustness of interventions to promote health and recovery among groups of participants, and investigate individual characteristics that may predict different responses to interventions. Planned analyses also include examination of service use and cost outcomes using administrative data in combination with interview results. Sensitivity analysis will be conducted to evaluate the effect of missing data using several methods, including mean substitution, multiple imputations, and last observation carried forward.

The results of narrative interviews will be analyzed according to the organizing themes of pathways into and out of homelessness, and high, low, and turning points in life. These thematic analyses are expected to complement results from questionnaires. *Post hoc* analyses will be informed by qualitative findings, and will also examine the characteristics of individuals who appear to exhibit better (and worse) outcomes regardless of randomization arm. In a related vein, ideographic analyses will be performed to examine whether CONG, HF-ACT, and TAU may be associated with better outcomes for differing subpopulations.

Administrative data will be analyzed to provide long-term (that is, up to 15 years prior to randomization) historical perspectives on trajectories of service use prior to and following homelessness. Administrative data will also be used as key outcome measures (for example, changes in hospitalization) and to validate specific items also collected via self-report (for example, hospitalized in past 6 months).

Data derived through the shared cross-site protocol are owned by the study sponsor. Data that are specific to VAH (unique instruments, administrative data) will be retained at the host institution (Simon Fraser University, Burnaby, BC, Canada). The use and storage of provincial administrative data are governed by Information Sharing Agreements between the Government of British Columbia and Simon Fraser University. The research protocol underwent institutional ethics review and was approved by Simon Fraser University.

## Results

**Figure 1:**
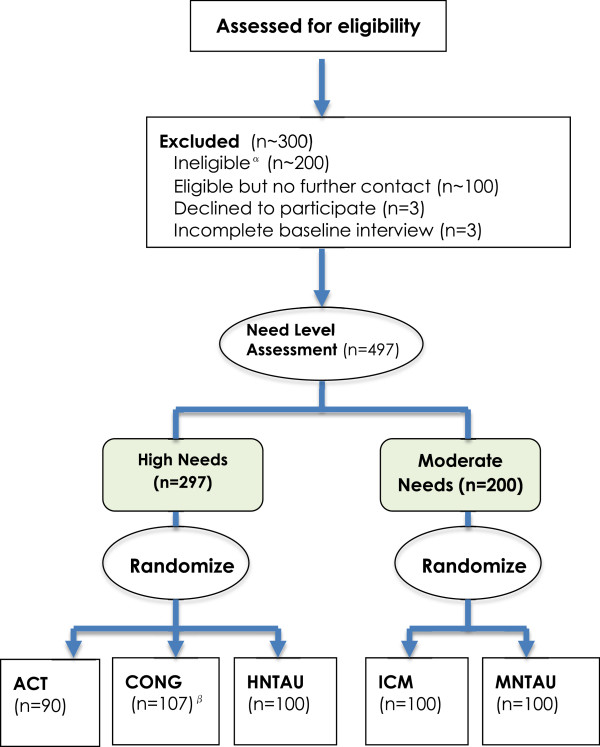
**Participant flow through eligibility, screening, needs level assessment, and allocation to study arm. **^α^Includes approximately 100 participants deemed ineligible via an informal telephone screen, and 94 participants who were ineligible after formal in-person screening. ^β^Includes 11 participants who were unable to be located after assignment or left within 1 month of entering.

Recruitment was carried out between October 2009 and April 2011. Follow-up interviews were completed on a schedule following each individual’s anniversary and were completed by May 2013. Approximately 800 individuals were screened by telephone. Referral sources (n = 40) represented about thirteen different types of services available to homeless adults with mental illness. The majority of potential participants were referred from homeless shelters, drop-in centers, homeless outreach teams, hospitals, community mental health teams, and criminal justice programs. Approximately 100 individuals were excluded via telephone due to clear ineligibility. A further 200 were excluded through the baseline interview procedure due to ineligibility (n = 94), unable to contact for baseline interview (n = 100), declining to participate (n = 3), or incomplete interview (n = 3) (Figure 1). Of the total number of participants randomized (n = 497), 200 met criteria for MN and 297 met criteria for HN. Retention rates by study arm after 24 months are illustrated in Table 2. Different rates are indicated in relation to scales administered every 3 months and every 6 months.

**Table 2:** Follow-up status for 'At Home’ participants after 24 months by need level

**Study arm**	**6 months questionnaire**					**3 months questionnaire**				
	**No follow-up**^**1**^				**At least one follow up**^**2**^	**No follow-up**^**3**^				**At least one follow up**^**4**^
	**Reason**			**Total (n = 23)**	**Total (n = 474)**	**Reason**			**Total (n = 16)**	**Total (n = 481)**
	**No contact**	**Death**^**5**^	**Withdrew consent**			**No contact**	**Death**^**6**^	**Withdrew consent**		
High Needs	5	5	-	10	287	3	3	-	6	291
CONG (n = 107)	1	1	-	2	105	-	-	-	-	107
ACT (n = 90)	-	1	-	1	89	-	-	-	-	90
HNTAU (n = 100)	4	3	-	7	93	3	3	-	6	94
Moderate Needs	7	4	2	13	187	6	2	2	10	190
ICM (n = 100)	2	1	-	3	97	2	0	-	2	98
MNTAU (n = 100)	5	3	2	10	90	4	2	2	8	92

^1^ - No follow-up data collected at any of 6, 12, 18 & 24 months.

^2^ - No significant differences between study arms was observed in terms of follow up completion rate (Fishers’ exact p values for HN & MN sample were 0.074 and 0.082 respectively).

^3^ - No follow-up data collected at any of 3, 6, 9, 12, 15, 18, 21 & 24 months visit.

^4^ - In terms of follow up completion rate, significant differences between study arms was observed for the HN sample, but no significant difference was observed for the MN sample (Fishers’ exact p values for HN & MN sample were 0.002 and 0.101 respectively).

^5^ - Total number of deaths (n = 29) was as follows: CONG-4; ACT-7; HNTAU-5; ICM-6; & MNTAU-7. However, 20 participants completed at least one follow up visit before death and the remaining 9 participants whose follow up data was not available died with seven months of randomization.

^6^ - Out of 29 deaths, 5 participants whose follow up data was not available died within five months of randomization and the rest 24 participants completed at least one follow up visit before death.

The primary reasons for loss to follow-up over 24 months were death (n = 5 for 3-month scales and n = 9 for 6-month scales) or inability to locate the participant (n = 9 for 3-month scales and n = 12 for 6-month scales). Some deaths occurred after participants had completed at least one follow-up interview and these data are eligible for analysis. The overall retention rate through 24 months was 97% (Table 2). No significant differences between study arms were observed in terms of follow-up (6-month scales) completion rates (Fisher’s exact *P* values for HN and MN samples were 0.074 and 0.082, respectively). For the 3-month scales, follow-up completion rates between HN study arms were significantly different (CONG, 100%; HF-ACT, 100%; TAU, 94%; Fisher’s exact *P* value = 0.002), but no significant difference was observed for the MN sample (HF-ICM, 98%; TAU, 92%; Fisher’s exact *P* value = 0.101). Of the 497 participants randomized, 438 (88%) gave consent to access administrative data from publicly-funded agencies.

Adverse events of all kinds were reported to a monitoring committee as well as to the Research Ethics Board at Simon Fraser University. Apart from mortality, adverse events typically involved episodes of interpersonal conflict, such as abusive language or offensive behavior involving participants.

Sociodemographic characteristics of participants are detailed in Table 3. Most were male (73%), white (56%), never married (70%), had a current medical illness (91%), were substance dependent (58%), and met criteria for 'absolute homelessness’ (78%).

**Table 3:** Socio-demographic and mental disorder related characteristics for the 'At Home’ participants by need status

**Variables**	**Overall N (%)**	**HN N (%)**	**MN N (%)**	**P value**
**Socio-demographics**				
Age at enrolment visit (years)				
Mean (SD)	40.8 (11.0)	39.7 (11.2)	42.6 (10.5)	**0.004**
Median (IQR)	41 (32–48)	39 (31–47)	44 (36–49)	**0.002**
Male Gender	359 (73)	218 (74)	141 (71)	0.420
Place of birth (Canada)	431 (87)	256 (87)	175 (88)	0.743
Ethnicity				
Aboriginals	77 (16)	44 (15)	33 (16)	0.844
White	280 (56)	170 (57)	110 (55)	
Other	140 (28)	83 (28)	57 (29)	
Incomplete High School	280 (57)	179 (61)	101 (51)	**0.022**
Single (never married)	343 (70)	214 (73)	129 (65)	**0.043**
Have children (under18)	122 (25)	69 (24)	53 (27)	0.483
Native Language (English)	392 (80)	236 (80)	156 (78)	0.696
**Homelessness**				
Precariously housed	109 (22)	65 (22)	44 (22)	0.976
Lifetime duration of homelessness (months)				
Mean (SD)	60.2 (70.3)	62.0 (67.0)	57.5 (74.9)	0.489
Median (IQR)	36 (12–84)	42 (12–84)	36 (12–84)	0.179
Longest duration of homelessness (months)				
Mean (SD)	30.9 (40.1)	32.2 (40.8)	28.9 (39.1)	0.358
Median (IQR)	12 (6–36)	18 (6–45)	12 (6–36)	0.236
Age of first homelessness (years)				
Mean (SD)	30.3 (13.3)	28.7 (12.5)	32.6 (14.1)	**0.002**
Median (IQR)	28 (19–41)	26 (19–36)	34 (20–44)	**0.003**
**Employment**				
Currently employed	18 (4)	10 (3)	8 (4)	0.722
Worked continuously (>1 year) in past	323 (65)	185 (63)	138 (69)	0.164
History of any wartime services	27 (5)	17 (6)	10 (5)	0.697
Willingness to have paid job	384 (87)	217 (84)	167 (90)	0.102
Hospitalized for mental illness (last 5 years)*				
Over 6 months	57 (12)	47 (16)	10 (5)	**<0.001**
More than two times	253 (53)	197 (69)	56 (29)	**<0.001**
**MINI International**				
**Neuropsychiatric Interview diagnosis**				
Psychotic Disorder/Schizophrenia*	263 (53)	211 (71)	52 (26)	**<0.001**
Major Depressive Episode	199 (40)	95 (32)	104 (52)	**<0.001**
Post Traumatic Stress Disorder (PTSD)	129 (26)	63 (21)	66 (33)	**0.003**
Manic or Hypomanic Episode*	97 (19)	68 (23)	29 (14)	**0.021**
Panic Disorder	104 (21)	59 (20)	45 (22)	0.479
Mood disorder with psychotic feature	84 (17)	56 (19)	28 (14)	0.152
Substance Dependence	288 (58)	183 (62)	105 (52)	**0.043**
Alcohol Dependence	121 (24)	72 (24)	49 (24)	0.948
Suicidality (high or moderate)	168 (34)	93 (31)	75 (37)	0.153
Two or more mental disorders	240 (52)	148(53)	92 (51)	0.402
Three or more mental disorders	114 (25)	78 (28)	36 (20)	**0.032**
**Referral sources**				
Shelter or transitional housing	143 (29)	82 (28)	61 (31)	**<0.001**
Housing Lists	19 (4)	9 (3)	10 (5)	
Outreach	86 (17)	44 (15)	42 (21)	
Hospitals	47 (9)	35 (12)	12 (6)	
Aboriginal groups	15 (3)	6 (2)	9 (4)	
Criminal justice	70 (14)	59 (20)	11 (6)	
Drop-in-centers	65 (13)	33 (11)	32 (16)	
Mental health teams	19 (4)	13 (4)	6 (3)	
Other	16 (3)	6 (2)	10 (5)	
Not specified	17 (3)	10 (3)	7 (3)	

* Variables used to determine eligibility for the HN sample.

A number of significant differences between the MN and HN samples were observed (Table 3 and 4). Several differences were expected based on inclusion criteria for each study and are reflected in the results. HN participants were more likely to have a psychotic disorder, have been hospitalized for psychiatric reasons, meet criteria for substance dependence, and have justice system involvement. HN participants also had lower MCAS scores.

**Table 4:** Questionnaire related characteristics for 'At Home’ participants by need status at enrolment visit

**Questionnaire**	**Overall mean (SD)**	**HN mean (SD)**	**MN mean (SD)**	**P value**
**Community Integration Scale (CIS)**				
Physical subscale score	2.1 (1.7)	1.9 (1.7)	2.4 (1.8)	**<0.001**
Psychological subscale score	10.9 (3.5)	11.0 (3.5)	10.7 (3.6)	0.368
**Colorado Symptom Index (CSI)**				
Total score	37.2 (12.5)	38.0 (13.1)	36.0 (11.7)	*0.098*
**Comorbid Conditions List (CMC)**^**1**^	**N (%)**	**N (%)**	**N (%)**	
Asthma	103 (21)	50 (17)	53 (26)	**0.009**
Hepatitis C	139 (30)	78 (28)	61 (31)	0.302
HIV/AIDS	43 (9)	18 (6)	25 (12)	**0.012**
Hepatitis B	25 (5)	13 (5)	12 (6)	0.412
Blood-borne infectious diseases^2^	157 (32)	87 (30)	70 (35)	0.224
Epilepsy or seizure	67 (13)	49 (16)	18 (9)	**0.016**
Stroke	27 (5)	19 (6)	8 (4)	0.248
Cancer	18 (4)	14 (5)	4 (2)	0.117
Head Injury	324 (65)	191 (64)	133 (67)	0.563
Presence of any physical illness	453 (91)	268 (90)	185 (93)	0.384
Multiple (≥ 2) physical illness	402 (81)	231 (78)	171 (86)	**0.032**
Multiple (≥ 3) physical illness	344 (69)	189 (64)	155 (78)	**0.001**
**EuroQuol 5D (EQ5D)**				
Overall health	61.0 (22.5)	61.8 (23.1)	60.0 (21.5)	0.382
**Food Security (FS)**				
Total score	4.6 (2.6)	4.5 (2.5)	4.8 (2.7)	0.214
**Global Assessment of Individual need –Substance Problem Scale (GAIN-SPS)**				
Total score (last month)	2.1 (2.0)	2.3 (2.0)	1.8 (2.0)	**0.007**
Age of first alcohol use	14.1 (6.3)	14.2 (5.0)	14.1 (4.9)	0.751
Age of first drug use	15.7 (5.0)	15.5 (5.6)	16.0 (7.2)	0.438
**Health Service Access Items (ACC)**				
Have a regular medical doctor	320 (65)	177 (60)	143 (72)	**0.008**
Place to go when you are sick	395 (81)	231 (79)	164 (83)	0.342
Needed health care, but didn’t receive it	209 (43)	129 (45)	80 (40)	0.269
**Health, Social Justice Service Use Inventory (HSJSU)**				
Seen by a health/social service provider	389 (79)	216 (74)	173 (89)	**<0.001**
Visited psychiatrist	134 (27)	89 (30)	45 (22)	*0.066*
Talked with a health/social service provider	112 (29)	58 (20)	54 (27)	*0.065*
Emergency room visit	281 (58)	163 (56)	118 (60)	0.483
Ambulance	195 (40)	118 (40)	77 (39)	0.748
Contacts with police (no arrest)	254 (52)	154 (53)	100 (51)	0.573
Held in a police cell (≤24 hours)	112 (23)	80 (28)	32 (16)	**0.002**
Arrested	173 (36)	128 (44)	45 (23)	**<0.001**
Court appearance	174 (36)	123 (43)	51 (26)	**<0.001**
**Interviewer Impression Items (III)**	**N (%)**	**N (%)**	**N (%)**	
Signs of difficulty in reading response card (a lot)	20 (4)	17 (6)	3 (1)	**0.019**
Signs of drug or alcohol intoxication (a lot)	10 (2)	7 (2)	3 (1)	0.505
Signs of psychiatric symptoms (a lot)	66 (13)	58 (19)	8 (4)	**<0.001**
Validity of information (no confidence)	14 (3)	13 (4)	1 (<1)	**0.010**
Multnomah Community Ability Scale (MCAS)*				
Total score	56.1 (9.6)	50.7 (6.8)	64.1 (7.3)	**<0.001**
**SF-12 Health Survey (SF-12)**				
Physical health	45.9 (12.3)	46.5 (12.4)	45.1 (12.2)	0.233
Mental health	35.4 (13.7)	35.8 (13.6)	34.8 (13.9)	0.445
**Quality of Life Index 20 Item (QOLI-20)**				
Total score	73.6 (21.9)	74.4 (21.5)	72.5 (22.4)	0.337
**Recovery Assessment Scale 22 item (RAS-22)**				
Total score	79.5 (12.0)	79.2 (11.5)	79.9 (12.8)	0.563
**Maudsley Addiction Profile (MAP)**	**N (%)**	**N (%)**	**N (%)**	
Use of alcohol	225 (46)	142 (48)	83 (42)	0.174
Use of heroin	96 (19)	59 (20)	37 (19)	0.732
Use of Cocaine	83 (17)	57 (19)	26 (13)	0.074
Use of Cocaine-crack base	160 (32)	97 (33)	63 (32)	0.805
Use of Amphetamine	61 (12)	44 (15)	17 (7)	**0.035**
Use of Cannabis	205 (45)	133 (47)	72 (42)	0.256
Injection drug use	88 (18)	54 (18)	34 (17)	0.727
Daily drug use (excluding alcohol)	126 (25)	82 (28)	44 (22)	0.159
Poly drug (≥ 3) use (excluding alcohol)	108 (22)	72 (24)	36 (18)	0.065

^1^ - Response 'Do not know’ was considered as no.

^2^ - Included HIV, Hepatitis C & Hepatitis B.

* Variables used to determine eligibility for the HN sample.

Beyond differences that were directly related to inclusion criteria (indicated with asterisk in Table 3 and Table 4), a number of additional significant differences between MN and HN were observed. MN participants were older at recruitment and when first homeless, were more likely to have been married, and more likely to have completed high school than those in the HN sample. Participants in the MN sample were more likely to report multiple physical illnesses, asthma, and HIV/AIDS than those in the HN sample.

Results of standardized questionnaires indicate broad similarities between the MN and HN samples (Table 4). No significant differences between groups were observed on measures of: community integration (CIS total score), health-related quality of life (EQ-5D), food security (FS), overall health (SF-12, physical or mental health scores), overall quality of life (QoLI-20), and personal recovery (RAS-22). The MN sample reported significantly greater physical integration in the community (CIS physical) and a significantly lower level of externalizing or substance-related needs (GAIN-SPS) than the HN sample.

A series of comparisons tested for potential non-equivalence between randomization arms at baseline. Sociodemographic and diagnostic characteristics for the three HN and two MN study arms are shown in Table 5. Within the HN sample, there were no significant baseline differences of sociodemographics and mental disorders between groups. In the MN sample, those randomized to TAU had longer durations of homelessness (*P* = 0.037) and were more likely to be absolutely homeless (*P* = 0.041) at the time of recruitment. Further comparisons of questionnaire results indicate no meaningful differences between randomization arms in either the MN or HN study, except for several comorbid medical conditions (HIV, hepatitis B, cancer). HN participants randomized to CONG had a significantly higher prevalence of HIV and hepatitis B, but when all blood-borne diseases (HIV, hepatitis B and C) were combined, no significant differences were observed between groups (Table 6).

**Table 5:** Comparisons of Socio-demographic and mental disorder related characteristics between study arms

**Variable**	**High need (n = 297)**				**Moderate need (n = 200)**		
	**CONG (n = 107) N (%)**	**ACT (n = 90) N (%)**	**TAU (n = 100) N (%)**	**P value**^**1**^	**ICM (n = 100) N (%)**	**TAU (n = 100) N (%)**	**P value**^**2**^
**Socio-Demographics**							
Age at randomization (years)							
Mean (SD)	40.0 (11.6)	39.5 (10.8)	39.5 (11.2)	0.910	42.1 (10.4)	43.1(10.6)	0.475
Median (IQR)	41 (30–48)	38 (31–47)	39 (32–48)	0.920	43 (34–50)	45 (36–49)	0.610
Male gender	82 (77)	66 (74)	70 (71)	0.696	71 (71)	70 (71)	0.964
Ethnicity							
Aboriginals	21 (20)	11 (12)	12 (12)	0.469	19 (19)	14 (14)	*0.060*
Caucasian	60 (56)	53 (59)	57 (57)		60 (60)	50 (50)	
Mixed/Other	26 (24)	26 (29)	31 (31)		21 (21)	36 (36)	
Incomplete high school	70 (66)	47 (53)	62 (62)	0.192	56 (56)	45 (45)	0.120
Single/Never married	76 (72)	63 (70)	75 (77)	0.591	66 (66)	63 (63)	0.658
Birth country (Canada)	94 (88)	80 (89)	82 (83)	0.417	90 (90)	85 (85)	0.285
Have children (under18)	24 (23)	21 (24)	24 (25)	0.938	28 (28)	25 (25)	0.692
Native Language (English)	87 (81)	76 (84)	73 (73)	0.125	13 (13)	16 (16)	0.733
**Homelessness**							
Absolutely homeless	88 (82)	72 (80)	72 (72)	0.179	72 (72)	84 (84)	**0.041**
Lifetime duration of homelessness (months)							
Mean (SD)	52.2 (63.5)	61.5 (69.1)	67.6 (69.0)	0.541	68.5 (92.1)	46.4 (50.7)	**0.037**
Median (IQR)	36 (12–72)	42 (12–84)	48 (13–109)	0.575	48 (14–93)	24 (12–72)	**0.051**
Longest duration of homelessness (months)							
Mean (SD)	32.9 (39.3)	30.5 (42.7)	33.0 (41.0)	0.901	31.7 (43.7)	26.0 (33.9)	0.304
Median (IQR)	20 (7–48)	12 (6–40)	12 (6–48)	0.623	48 (6–40)	12 (5–34)	0.321
Age of first homelessness (years)							
Mean (SD)	29.9 (13.1)	28.0 (11.9)	28.0 (12.4)	0.474	30.8 (13.8)	34.3 (14.3)	*0.083*
Median (1QR)	27 (20–39)	26 (19–35)	24 (18–36)	0.463	29 (18–42)	35 (21–45)	0.118
**Employment**							
Currently employed	5 (5)	1 (1)	4 (4)	0.368*	6 (6)	2 (2)	0.279*
Worked continuously (>1 year) in past	66 (62)	58 (69)	61 (61)	0.773	66 (66)	72 (72)	0.987
Wartime services in past	4 (4)	7 (8)	6 (6)	0.448	5 (5)	5 (5)	0.987
Willingness to have paid job	79 (82)	68 (90)	70 (82)	0.353	80 (89)	87 (91)	0.696
**Hospitalized for mental illness (last 5 years)**							
Over 6 months	14 (13)	18 (20)	15 (15)	0.406	4 (4)	6 (6)	0.506
More than two times	73 (70)	57 (68)	67 (71)	0.927	25 (25)	31 (32)	0.299
**MINI International Neuropsychiatric Interview diagnosis**							
Major Depressive Episode	35 (33)	31 (34)	29 (29)	0.710	52 (52)	52 (52)	1.00
Manic or Hypomanic Episode	25 (23)	23 (26)	20 (20)	0.654	11 (11)	18 (18)	0.160
Post-Traumatic Stress Disorder	27 (25)	17 (19)	19 (19)	0.445	34 (34)	32 (32)	0.802
Panic Disorder	20 (19)	15 (17)	24 (24)	0.418	19 (19)	26 (26)	0.236
Mood Disorder with psychotic feature	20 (19)	17 (19)	19 (19)	0.997	16 (16)	12 (12)	0.415
Psychotic Disorder	79 (74)	59 (66)	73 (73)	0.385	25 (25)	27 (27)	0.747
Alcohol dependence	28 (26)	19 (21)	25 (25)	0.695	25 (25)	24 (24)	0.869
Substance dependence	67 (63)	55 (61)	61 (61)	0.965	51 (51)	54 (54)	0.671
Suicidality (moderate or high)	34 (32)	28 (31)	31 (31)	0.992	33 (33)	42 (42)	0.189
Two or more mental disorders	53 (49)	41 (46)	54 (54)	0.507	44 (44)	48 (48)	0.547
Three or more mental disorders	34 (32)	22 (24)	22 (22)	0.250	17 (17)	19 (19)	0.713
**Referral sources**							
Shelter or transitional housing	31 (29)	26 (29)	25 (25)	0.468	31 (31)	30 (30)	0.250
Housing Lists	3 (3)	4 (4)	2 (2)		6 (6)	4 (4)	
Outreach	13 (12)	15 (17)	16 (16)		21 (21)	21 (21)	
Hospitals	11 (10)	11 (12)	13 (13)		4 (4)	8 (8)	
Aboriginal groups	2 (2)	2 (2)	2 (2)		6 (6)	3 (3)	
Criminal justice	27 (25)	14 (16)	18 (18)		5 (5)	6 (6)	
Drop-in-centers	15 (14)	9 (10)	9 (6)		20 (20)	12 (12)	
Mental health teams	4 (4)	3 (3)	6 (6)		0 (0)	6 (6)	
Other	0 (0)	1 (1)	5 (5)		4 (4)	6 (6)	
Not specified	1 (1)	5 (6)	4 (4)		3 (3)	4 (4)	

^1^ - Bold indicates p value ≤ 0.05 and Italic indicates p value between > 0.05 and ≤ 0.1.

^2^ - Bold indicates p value ≤ 0.05 and Italic indicates p value between > 0.05 and ≤ 0.1.

* - P value from Fisher’s Exact Test.

**Table 6:** Comparisons of questionnaire related characteristics between study arms at enrolment visit

**Questionnaire**	**High need (n = 297)**				**Moderate need (n = 200)**		
	**CONG (n = 107) N (%) or Mean (SD)**	**ACT (n = 90) N (%) or Mean (SD)**	**TAU (n = 100) N (%) or Mean (SD)**	**P value**^**1**^	**ICM (n = 100) N (%) or Mean (SD)**	**TAU (n = 100) N (%) or Mean (SD)**	**P value**^**2**^
**Community Integration Scale (CIS)**							
Physical subscale score	2.1 (1.8)	1.6 (1.5)	1.8 (1.7)	0.148	2.5 (1.8)	2.4 (1.7)	0.884
Psychological subscale score	10.6 (3.7)	11.3 (3.5)	11.1 (3.2)	0.384	10.4 (3.5)	11.0 (3.6)	0.241
**Colorado Symptom Index (CSI)**							
Total score	37.1 (13.0)	36.4 (13.4)	40.2 (12.6)	*0.093*	36.1 (12.2)	36.0 (11.2)	0.954
**Comorbid Conditions List (CMC)**^**3**^	**N (%)**	**N (%)**	**N (%)**		**N (%)**	**N (%)**	
Asthma	18 (17)	14 (16)	18 (18)	0.904	27 (27)	26 (26)	0.873
Hepatitis C	26 (24)	23 (26)	29 (29)	0.732	32 (32)	29 (29)	0.645
HIV/AIDS	12 (11)	2 (2)	4 (4)	0.025*	16 (16)	9 (9)	0.134
Hepatitis B	9 (8)	1 (1)	3 (3)	0.039*	7 (7)	5 (5)	0.552
Blood-borne infectious diseases^4^	33 (32)	23 (26)	31 (32)	0.572	37 (37)	33 (33)	0.553
Epilepsy or seizure	20 (19)	10 (11)	19 (19)	0.256	5 (5)	13 (13)	0.081*
Stroke	11 (10)	2 (2)	6 (6)	0.069	2 (2)	6 (6)	0.279*
Cancer	4 (4)	1 (1)	9 (9)	0.036*	0 (0)	4 (4)	0.058*
Head Injury	66 (62)	62 (69)	63 (63)	0.544	61 (61)	72 (73)	*0.079*
Presence of any physical illness	98 (92)	81 (90)	89 (89)	0.818	90 (90)	95 (95)	0.179
Multiple (≥ 2) physical illness	82 (77)	69 (77)	80 (80)	0.806	84 (84)	87 (87)	0.547
Multiple (≥ 3) physical illness	69 (65)	52 (58)	68 (68)	0.334	78 (78)	77 (77)	0.866
**EuroQuol 5D (EQ5D)**							
Overall health	59.5 (23.7)	64.2 (22.9)	62.0 (22.5)	0.361	58.4 (23.4)	61.5 (19.5)	0.325
**Food Security (FS)**							
Total score	4.3 (2.6)	4.4 (2.6)	4.8 (2.4)	0.454	4.7 (2.8)	4.8 (2.7)	0.799
**Global Assessment of Individual need –Substance Problem Scale (GAIN-SPS)**							
Total score (last month)	2.4 (2.0)	2.1 (1.9)	2.3 (2.0)	0.591	1.8 (2.0)	1.8 (2.0)	0.842
Age of first alcohol use	14.3 (6.5)	14.2 (3.8)	14.2 (4.0)	0.979	13.8 (4.8)	14.3 (4.9)	0.523
Age of first drug use	15.6 (6.5)	15.3 (5.0)	15.7 (5.0)	0.892	15.4 (6.3)	16.5 (7.9)	0.304
**Health Service Access Items (ACC)**							
Have a regular medical doctor	64 (60)	53 (59)	60 (61)	0.971	71 (71)	72 (72)	0.876
Place to go when you are sick	82 (80)	72 (81)	77 (79)	0.913	83 (84)	81 (82)	0.706
Needed health care, but didn’t receive it	45 (43)	36 (41)	48 (51)	0.371	39 (39)	41 (41)	0.817
**Health, Social Justice Service Use Inventory (HSJSU)**	**N (%)**	**N (%)**	**N (%)**		**N (%)**	**N (%)**	
Seen a health/social service provider	70 (66)	67 (75)	79 (80)	*0.075*	84 (84)	89 (90)	0.217
Visited psychiatrist	28 (26)	27 (30)	34 (34)	0.470	20 (20)	25 (25)	0.397
Talked a health/social service provider	12 (20)	18 (20)	19 (20)	0.992	31 (31)	23 (23)	0.218
Emergency room visit	59 (57)	51 (57)	53 (54)	0.912	64 (64)	54 (55)	0.202
Ambulance	47 (44)	33 (37)	38 (38)	0.535	35 (35)	42 (38)	0.282
Contacts with police (no arrest)	53 (52)	42 (47)	59 (60)	0.226	45 (45)	55 (56)	0.118
Held in a police cell (≤24 hours)	25 (24)	27 (31)	28 (30)	0.556	14 (14)	18 (19)	0.386
Arrested	50 (48)	32 (36)	46 (48)	0.152	19 (19)	26 (27)	0.179
Court appearance	51 (50)	34 (38)	38 (40)	0.189	22 (22)	29 (30)	0.222
**Interviewer Impression Items (III)**	**N (%)**	**N (%)**	**N (%)**		**N (%)**	**N (%)**	
Signs of difficulty in reading card (a lot)	6 (6)	6 (7)	5 (5)	0.883	2 (2)	1 (1)	1.00^*^
Signs of drug or alcohol intoxication (a lot)	1 (1)	2 (2)	4 (4)	0.349^*^	1 (1)	2 (2)	1.00^*^
Signs of psychiatric symptoms (a lot)	25 (23)	11 (12)	22 (22)	0.108	4 (4)	4 (4)	1.00^*^
Validity of information (no confidence)	7 (6)	4 (4)	2 (2)	0.287^*^	1 (1)	0 (0)	1.00^*^
**Multnomah Community Ability Scale (MCAS)**							
Total score	49.9.0 (6.7)	51.6 (6.5)	50.6 (7.0)	0.195	64.1 (7.6)	64.1 (7.1)	0.962
**SF-12 Health Survey (SF-12)**							
Physical health	47.4 (13.1)	46.7 (12.3)	45.3 (11.6)	0.466	43.9 (12.1)	46.4 (12.9)	0.140
Mental health	34.8 (15.1)	36.9 (13.0)	35.8 (12.6)	0.551	35.7 (13.0)	33.9 (14.8)	0.371
**Quality of Life Index 20 Item (QOLI-20)**							
Total score	72.6 (21.7)	76.2 (21.3)	74.7 (21.4)	0.497	72.2 (21.6)	72.8 (23.3)	0.851
**Recovery Assessment Scale 22 item (RAS-22)**							
Total score	78.2 (12.1)	80.7 (11.5)	79.1 (10.7)	0.308	80.3 (11.3)	79.5 (14.1)	0.685
**Maudsley Addiction Profile (MAP)**	**N (%)**	**N (%)**	**N (%)**		**N (%)**	**N (%)**	
Use of alcohol	50 (47)	44 (49)	48 (49)	0.936	37 (38)	46 (46)	0.240
Use of heroin	24 (22)	13 (14)	22 (22)	0.295	18 (18)	19 (19)	0.909
Use of Cocaine	24 (22)	14 (16)	19 (19)	0.476	12 (12)	14 (14)	0.715
Use of Cocaine-crack base	36 (34)	26 (29)	35 (36)	0.596	36 (37)	27 (27)	0.141
Use of Amphetamine	12 (11)	17 (18)	15 (15)	0.346	10 (10)	7 (7)	0.421
Use of Cannabis	46 (45)	40 (48)	47 (49)	0.848	35 (42)	37 (42)	0.960
Injection drug use	19 (18)	16 (18)	19 (20)	0.944	18 (18)	16 (16)	0.682
Daily drug use (excluding alcohol)	31 (29)	19 (21)	32 (32)	0.227	27 (27)	17 (17)	*0.088*
Poly drug (≥ 3) use (excluding alcohol)	30 (28)	17 (29)	25 (25)	0.318	20 (20)	16 (16)	0.421

^1^ -Bold indicates p value ≤ 0.05 and Italic indicates p value between > 0.05 and ≤ 0.1.

^2^ -Bold indicates p value ≤ 0.05 and Italic indicates p value between > 0.05 and ≤ 0.1.

^2^ -Response 'Do not know’ was considered as no.

^4^ -Included HIV, Hepatitis C & Hepatitis B.

* -P value from Fisher’s Exact Test.

## Discussion

As expected, inclusion criteria led to a number of significant differences between the MN and HN samples. Compared to those in MN, members of the HN cohort were significantly more likely to meet criteria for psychosis or mania/hypomania, have multiple recent psychiatric hospitalizations, and be severely compromised in their community functioning. Other significant differences between MN and HN were not directly related to the inclusion/exclusion criteria. Members of the HN cohort had lower educational achievement and were more likely to have multiple mental disorders than those assigned to the MN study. In numerous other respects, participants assigned to MN and HN did not differ significantly. Overall, participants were white, male, 'absolutely homeless’, physically ill, and met criteria for substance dependence and alcohol dependence.

Participants assigned to MN were significantly more likely to meet criteria for post-traumatic stress disorder, major depression, and report having HIV/AIDS than those assigned to HN. Thirty percent of the MN cohort had been homeless for more than 60 months in their lifetime and 26% met criteria for a psychotic disorder. These results suggest that the descriptor 'moderate’ is a misnomer that understates the complexity of needs within the MN cohort. As hypothesized, the profile of both study cohorts (MN and HN) included level of needs (for example, substance dependence, physical illness) that have not been included in previous studies of HF. VAH is therefore capable of generating new knowledge regarding the effectiveness of HF-ICM and HF-ACT for clients with a broad range of presenting characteristics.

Randomization successfully minimized differences between study arms. We tested for differences on sociodemographic and mental health-related variables, as well as all other measures (total score or subscale score or individual item) administered at baseline. In the MN study, those assigned to the TAU arm were significantly more likely to be 'absolutely homeless’ and to have lower lifetime duration of homelessness. No other significant differences were observed in either study, except for several comorbid medical conditions (HIV, hepatitis B, cancer) with very low prevalence. HN participants randomized to CONG had a significantly higher prevalence of HIV and hepatitis B, but when all blood-borne diseases (HIV, hepatitis B and C) were combined, no significant differences were observed between groups. Despite differences between groups, the low prevalence of these conditions in the sample is not expected to influence results. However, tests will be designed to control for relevant differences identified at baseline. Previous research has indicated that homeless mentally ill individuals may have a preference for independent apartments over group housing when offered a choice [83], and that these preferences may change based on experiences after the initiation of housing [84]. In the present study, individuals randomized to HF-ACT had a choice of apartments, but those randomized to CONG were limited to selecting from among the available units in one building. Narrative interviews will be examined in order to assess whether participants experienced meaningful differences concerning their choice of housing in either of these two settings, and whether the experience of choice was related to outcomes of interest.

A high percentage of participants in each study arm (92% to 100%) were successfully followed through 24 months of interviews. Twenty-nine participants died during the 24 months following randomization. Unsurprisingly, participants assigned to TAU were most likely to be lost to follow-up during the 24 months post-randomization. However, the differences were not statistically significant. Differences in follow-up for the 3-month scales were significant in the HN study only. Despite statistically significant differences, the high overall follow-up rate in each group (94% in one arm, 100% in two others) is expected to yield valid and generalizable results. This high follow-up rate is attributable to diverse strategies, including extensive outreach, a welcoming field office, relationships with service providers in the field, and maintaining updated information regarding collateral contacts and daily routines. More generally, the recruitment and retention of a knowledgeable and committed team of interviewers is a critical factor.

VAH shares several important methodological features with studies in four other Canadian sites, primarily: inclusion/exclusion criteria, randomization to HF or usual care, a common battery of cross-site measures, and semi-structured qualitative interviews with a subset of participants [1]. At the same time, the current study has a number of important site-specific elements. VAH is the only RCT to compare different configurations of HF (congregate and scattered sites) alongside TAU. The results of this comparison will offer guidance to many cities, including Vancouver, that include congregate variations of HF as part of their strategies to address homelessness [85]. In addition, this trial has recruited samples with a broader range and severity of symptoms than those reflected in previous studies. For example, the high prevalence of substance use disorders in this study will help to fill a specific gap in knowledge regarding the robustness of HF for individuals with concurrent disorders [86].

A number of unique measures were incorporated in the protocol based on their expected relevance to the local population, including measures of addiction frequency and severity, neuropsychological functioning, and physical examinations. In addition, VAH incorporates a large array of administrative data spanning diverse publicly-funded services and interventions across time. These data generate opportunities to study the trajectories of service involvement over 10 years prior to recruitment in VAH, and enable follow-up of participants after the completion of the intervention. The inclusion of longitudinal data from multiple relevant sectors (justice, health, financial assistance) provides a unique opportunity for cost-based analyses.

This study combines models of housing with support in each of the intervention conditions. This may make it difficult to isolate the relative contribution of distinct components (for example, housing alone) that best account for any observed differences or improvements. Each of the data sources included in the VAH protocol is subject to sources of bias. Several questionnaires have not been well validated with homeless mentally ill samples, and a small number were adapted or developed for the present study. Narrative interviews with selected participants may yield findings that are unrepresentative. Administrative data sources are subject to limitations that include the ability to match all subjects across all databases, and the accuracy and completeness of the resulting extracts. However, the combination of administrative data, narrative interviews, and questionnaires enables the application of mixed-methods approaches that enrich understanding beyond the scope of each individual data type. Finally, while other sites abbreviated their study durations to 21 months, VAH maintained the original 24-month protocol, and therefore preserved a greater opportunity to detect changes that may require a longer period of observation.

## Conclusions

The present results confirm that VAH has successfully implemented experimental protocols that promise to generate new knowledge regarding interventions for individuals who are both homeless and mentally ill. Participants were successfully recruited and retained through the follow-up period, and randomization effectively minimized differences between study arms in each trial. Diverse data sources and relatively long follow-up provide opportunities for multi-method approaches to longitudinal data analysis. VAH adds to previous research on HF by including a sample with complex comorbidities and concurrent substance use disorders, and is the first experiment to include congregate housing alongside scattered site HF.

## Abbreviations

ACC: Health service access items; ACE: adverse childhood experiences; ACT: Assertive community treatment; ANOVA: Analysis of variance; CI: Cognitive impairment; CIS: Community integration scale; CMC: Comorbid conditions list; CONG: Congregate housing with support; CONSORT: Consolidated standards of reporting trials; CSI: Colorado symptom index (modified); C-SSS: Core service satisfaction scale; CTS: Conflict tactics scale; EQ-5D: EuroQol 5D; FS: Social support items and food security; GAIN-SPS: Global appraisal of individual needs, substance problem scale; GEE: Generalized estimating equation; HF: Housing first; HLM: Hierarchical linear modeling; HN: High needs; HSJSU: health, social, and justice service use inventory; ICM: Intensive case management; LR: Landlord relations; MAP: Maudsley addiction profile; MCAS: Multnomah community ability scale; MH: Mobility history; MINI: Mini-international neuropsychiatric interview; MN: Moderate needs; MoCA: Montreal cognitive assessment; OHQS: Observer-rated housing quality scale; PHQL: Perceived housing quality; QoLI-20: Quality of life index, 20-item; RAS-22: Recovery assessment scale, 22-item; RCT: Randomized controlled trial; RTLFB: Residential time-line follow-back; SF-12: SF-12 health survey; SRO: Single room occupancy; TAU: Treatment as usual; VAH: Vancouver at home; VFC: Foster care history; VTLFB: Vocational time-line follow-back; WAI: Working alliance inventory.

## Competing interests

The authors declare that they have no competing interests.

## Authors’ contributions

JMS is lead investigator of the study, and drafted and finalized the manuscript. MLP oversaw field research and wrote sections of the manuscript. AM conducted statistical analyses and prepared results for the manuscript. LC administered interviews and prepared sections of the manuscript. SNR contributed to data analysis and prepared sections of the manuscript. AP contributed to the design of the study and edited the manuscript. KF contributed to the design and implementation of the study. All authors read and approved the final manuscript.
